# Novel codons in rat Pdx-1 complementary DNA

**DOI:** 10.1186/s13104-018-3837-0

**Published:** 2018-10-12

**Authors:** Takayoshi Kiba

**Affiliations:** Department of Life Sciences, Faculty of Science, Okayama University of Science, 1-1 Ridai-cho, Kita-ku, Okayama-shi, Okayama 700-0005 Japan

**Keywords:** cDNA, Pdx-1, Rat

## Abstract

**Objectives:**

Pancreatic and duodenal homeobox-1 (Pdx-1) is a homeodomain-containing transcription factor essential for pancreatic development, beta-cell differentiation and the maintenance of mature beta cell function. To transfect the expression vectors of Pdx-1 in the mammalian cells, the complementary DNA (cDNA) of Pdx-1 was conducted.

**Results:**

Novel codons and amino acids sequences were detected in rat Pdx-1 cDNA. Comparing the previous reports regarding rat Pdx-1 cDNA, 3 novel codons (ACA141CCA, AAG720CCG, GTT742GCT) were detected. The amino acids sequences based on the detected cDNA sequences confirmed those, which were already available in public databases. The present study described novel codons in rat Pdx-1 cDNA. The results may be useful for an effective research against pancreatic development, regeneration or carcinogenesis regarding Pdx-1 expressions.

## Introduction

Pancreatic and duodenal homeobox-1 (Pdx-1) is known to be a homeodomain-containing transcription factor for pancreatic development, beta-cell differentiation and the maintenance of mature beta cell function by regulating expressions of many key endocrine beta-cell-specific genes [1]. Also, Pdx-1 directly controls insulin gene expression [2] and the expression of the genes encoding glucose transporter 2 (Slc2a2*)* [3], islet amyloid polypeptide precursor [4], Pax 4 [5], synaptotagmin 1 [6], and Pdx-1 itself [7].

Rat chromosome 12 is associated with Pdx-1 gene, which shares a 88% amino acid homology with human [8]. Rat Pdx-1 has two exons and it is a protein of 283 amino acids with a calculated molecular weight of 30.83 kDa. According to the functional domains and phosphorylation sites of human PDX-1, it has been reported that the sequences of 11, 61, 66, 151, 231 and 232 amino acids sites, 1–79 amino acids sites, 146–206 amino acids sites, 188–203 amino acids sites, 191–196 amino acids sites and 197–203 amino acids sites are related with phosphorylation sites, transactivation sites, homeobox sites, protein transduction domain, DNA-binding motif and nuclear localization signal of Pdx-1, respectively [8].

To transfect the expression vector of Pdx-1 in the mammalian cells, when the complementary DNA (cDNA) of Pdx-1 was conducted, the detected sequences were different from those reported before [9] (https://www.ncbi.nlm.nih.gov/nuccore/454391). In this report, the author has reported novel codons in rat Pdx-1 cDNA.

## Main text

### Materials and methods

#### Animals

A female Wistar rat weighing 140 g (age in 6 weeks) were used in this study. It was maintained in a temperature- and light-controlled environment (23 ± 2 °C; 12-h light/12-h dark cycle) and were given free access to food and water. A rat was euthanized by cervical dislocation under anesthesia with medetomidine (0.75 mg/kg), midazolam (4 mg/kg), and butorphanol tartrate (5 mg/kg) by intraperitoneal route for the following experiments.

#### Total RNA Preparation and cDNA synthesis

In the present study, total RNA was isolated from fresh pancreatic tissue. The author previously described a technique that reliably improves the amount and the quality of RNA extracted from rat pancreas, an RNase-rich organ, using RNAlater-ICE [10]. RNA integrity was confirmed by agarose gel electrophoresis. Total RNA was reverse transcribed using PrimeScript™ Double Strand cDNA Synthesis Kit (Takara Bio Inc., Kusatsu, Japan). Synthesis of first strand cDNA was performed with oligo (dT) 18 primer and random hexamer primers simultaneously. Oligo (dT) 18 primers synthesize cDNA from the poly (A) tail mRNA, while random primers initiate cDNA synthesis from rest of the RNA population.

#### cDNA cloning of rat Pdx-1

Primers were designed to the 5′ and 3′ ends of rat cDNA based on the sequence from GenBank accession number NC_005111.4. The forward and reverse primers were: 5′ TCCGCTAGCCACCATGAATAGTGAGGAGCA 3′ and 5′ TTCGAAGCTTAAATCACCGGGGTTCCTGCGGT 3′, respectively. These primers were used to PCR amplify coding sequence of Pdx1 from a cDNA library originated from a rat pancreas RNA and a rat pancreas QUICK-Clone™ cDNA library (Clontech Laboratories, Mountain View, CA, USA). *Nhe*-I and *Hind*-III sites were incorporated into the primers at the 5′ and 3′ ends, respectively, to allow sub-cloning into the pEGFP-N1 mammalian expression vector (Clontech Laboratories). cDNA at Pdx-1, 852 base pairs (bp), was cloned by polymerase chain reaction (PCR) amplification, using PrimeSTAR^®^ HS DNA Polymerase (Takara Bio Inc.) and KOD-Plus-Neo^®^ (TOKOBO. Inc., Osaka, Japan). Polymerase Chain Reaction (PCR) was accomplished in a microtube containing 5 μL of 10× PCR buffer, 5 μL of dNTPs (0.2 mM for each), 3 μL of MgCl_2_ (1.5 mM), 1 μL of each primer with the concentration of 10 µM for each, 1–2 μL of template DNA, 1 unit of PrimeSTAR^®^ HS DNA Polymerase or KOD-Plus-Neo^®^ and nuclease-free ddH_2_O up to 50 μL final volume. Amplification reactions were performed in ASTEC thermocycler (Shime, Japan) and the PCR program included the following steps for all the amplicons [94 °C: 2 min, followed by 45 cycles of denature 98 °C: 10 s; extension 68 °C: based on 30 s for each kbp]. PCR products were evaluated by electrophoresis using 1% (w/v) agarose gel. Sequencing was carried out on a 3730xl DNA Analyzer (Thermo Fisher Scientific, Tokyo, Japan) at Eurofins Genomics (Tokyo, Japan).

### Results and discussion

**Fig. 1:**
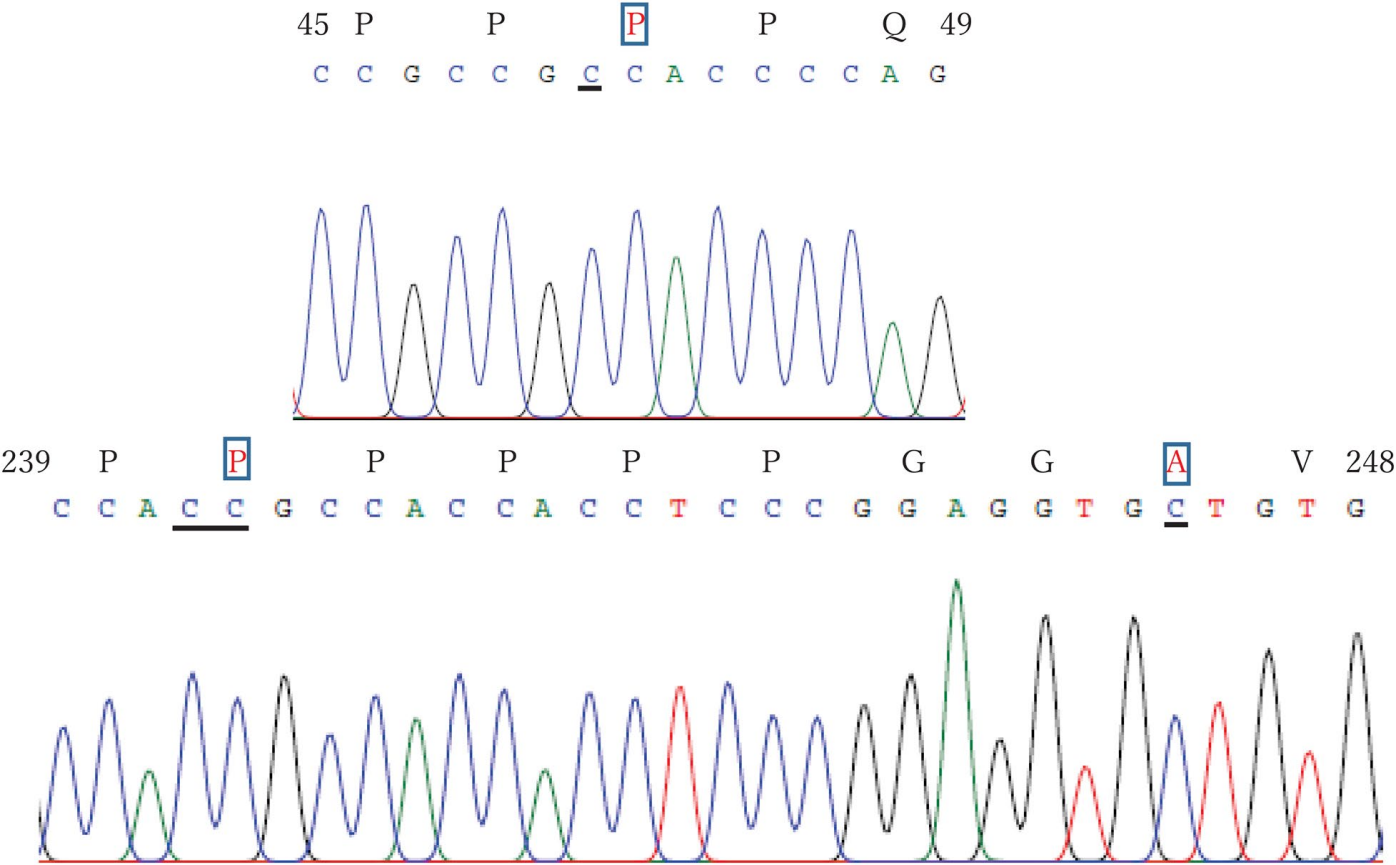
The novel codons and amino acids sequence in Pdx-1 are shown. The underline regions mean the novel cDNA sequences, and the open square regions mean the amino acids sequence, which is related with the detected cDNA sequences in the present study

**Fig. 2:**
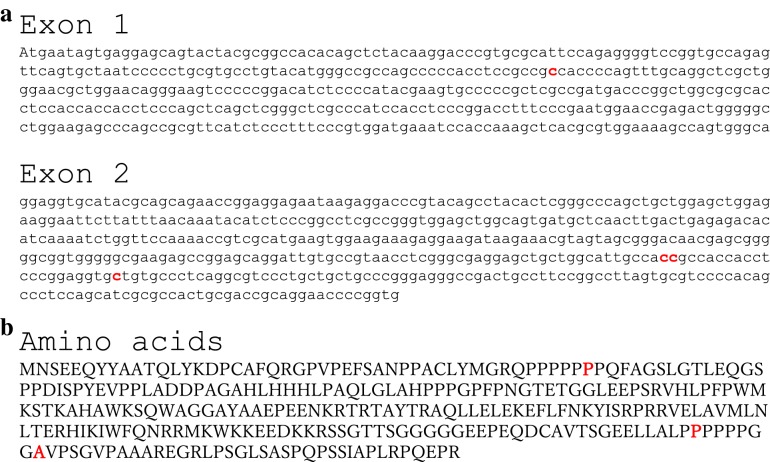
The correct nucleic acids in Exon 1 and 2 (**a**) and the amino acids sequence (**b**) of Pdx-1, which are already available in public databases, are shown. In **a**, the red collar regions mean the novel cDNA sequences and in **b**, the the red collar regions mean the amino acids sequence, which is related with the detected cDNA sequences in the present study

In the present study, the author used two different cDNA (a cDNA library originated from a rat pancreas RNA and a rat pancreas QUICK-Clone™ cDNA library) and two different PCR enzyme (PrimeSTAR^®^ HS DNA Polymerase and KOD-Plus-Neo^®^). These methods indicated the same results regarding Pdx-1 cDNA (Figs. 1, 2). Comparing the previous reports regarding rat Pdx-1 cDNA [9] (https://www.ncbi.nlm.nih.gov/nuccore/454391), 3 novel codons (ACA141CCA, AAG720CCG, GTT742GCT) were detected. These 3 novel codons were confirmed with genomic DNA, not cDNA, in rat chromosome 12p11, which was already reported before [11] (https://www.ncbi.nlm.nih.gov/nuccore/NC_005111.4?report=genbank&from=9496044&to=9501211&strand=true). In the present study, rat Pdx-1 is a protein of 283 amino acids with a calculated molecular weight of 30.83 kDa, using Compute pI/Mw tool (https://web.expasy.org/compute_pi/). Rat Pdx-1 shares a 90% amino acid homology with human, using Web BLAST (https://blast.ncbi.nlm.nih.gov/Blast.cgi). The amino acids sequences based on the detected cDNA sequences also confirmed those, which are already available in public databases (GenBank: EDL89565.1 and UniProtKB/Swiss-Prot: P52947.1) (Fig. 2). Therefore, the author speculates that the previous report regarding the nucleotide sequences in rat Pdx-1 cDNA is incorrect, because the detected sequences of the cDNA of Pdx-1 were different from those reported before [9] (https://www.ncbi.nlm.nih.gov/nuccore/454391).

### Conclusion

The present study described novel codons in rat Pdx-1 cDNA. The results may be useful for an effective research against pancreatic development, regeneration or carcinogenesis regarding Pdx-1 expressions.

### Limitations

In the present study, novel codons and amino acids sequences were detected in rat Pdx-1 cDNA. Comparing the previous reports regarding rat Pdx-1 cDNA (https://www.ncbi.nlm.nih.gov/nuccore/454391), 3 novel codons (ACA141CCA, AAG720CCG, GTT742GCT) were detected. However, these 3 novel codons were confirmed with genomic DNA, not cDNA, in rat chromosome 12p11, which was already reported before (https://www.ncbi.nlm.nih.gov/nuccore/NC_005111.4?report=genbank&from=9496044&to=9501211&strand=true). Also, the amino acids sequences based on the detected cDNA sequences confirmed those, which were already available in public databases.

## Abbreviations

cDNA: complementary DNA
PCR: polymerase chain reaction
Pdx-1: pancreatic and duodenal homeobox 1
